# Abusive head trauma: a consensus statement on a shared workflow model within the Balkan network for quality improvement in forensic medicine

**DOI:** 10.3389/fped.2026.1800421

**Published:** 2026-04-20

**Authors:** Monica Concato, Maximo Leonardo Franzoni, Tijana Petrović, Anita Galic, Viktorija Belakaposka Srpanova, Emina Dervisevic, Chara Spiliopoulou, Stefano D’Errico

**Affiliations:** 1 Department of Medical Surgical and Health Sciences, University of Trieste, Trieste, Italy; 2 Institute of Forensic Medicine ‘Milovan Milovanovic’, Faculty of Medicine, University of Belgrade, Belgrade, Serbia; 3 School of Medicine, University of Zagreb, Zagreb, Croatia; 4 Institute for Forensic Medicine, Criminalistic and Medical Deontology, Medical Faculty, Skopje, North Macedonia; 5 Department of Forensic Medicine, Faculty of Medicine, University of Sarajevo, Sarajevo, Bosnia and Herzegovina; 6 Department of Forensic Medicine and Toxicology, School of Medicine, National and Kapodistrian University of Athens, Athens, Greece

**Keywords:** AHT, autopsy, NAHI, quality standard, SBS

## Abstract

Abusive head trauma (AHT), is considered a leading cause of fatalities resulting from physical abuse in infants under 2 years of age, with a peak incidence between 1 and 2 months after birth. The incidence of AHT ranges from 14 to approximately 40 cases per 100,000 children in industrialized countries with a mortality rate ranging from 10 to 20%. The absence of internationally recognized best practices or guidelines especially in the field of forensic medicine has resulted in methodological variability in the management of these cases across different settings. In response to this gap, a comparative working group involving experts from Italy and the Balkan countries was established, leading to the creation of a shared discussion platform. The aim of this collaborative effort was to identify strengths and critical issues in the forensic handling of abusive head trauma, ultimately with the goal of developing a shared workflow chart for the management of these complex cases within the network.

## 1 Introduction

Abusive head trauma (AHT) has an incidence rate ranging from 14 to 40 cases per 100,000 children in industrialized countries (1), and 25 to 35 children per 100,000 under 1 year of age in the United States (2). The term ‘abusive head trauma’ has superseded the older definition of ’shaken baby syndrome’ (SBS), encompassing the full spectrum of potential injurious mechanisms, including violent shaking as well as deliberate impact resulting in acceleration–deceleration forces, head compression, penetrating head injury or combined rotation and impact, and whiplash-type mechanisms (3, 4).

However, it is suspected that the phenomenon is much more widespread and remains largely unrecognized due to the lack of up-to-date epidemiological data (1) and the considerable diagnostic challenges (3). Moreover, it represents the leading cause of death in children under two years of age (3) with a peak of incidence between 1 and 2 months after birth (5). While more than half sustain severe neurological injuries and/or permanent visual impairments, approximately one in four victims die (6).

Although no universally accepted best-practice approach currently exists for clinical diagnosis of child abuse, the study by Blangis et al. (7) reviewed 20 guidelines encompassing 15 countries between 2010 and 2020. The literature strongly highlights the crucial role of autopsy in the diagnosis of abuse (8–11), dedicated protocols are scarce and frequently sector-specific, addressing individual components such as ocular investigation (2).

## 2 Methods

### 2.1 Study design and setting

This study was conceived as a multicentric, qualitative, comparative methodological investigation conducted within a collaborative network of Institutes of Forensic Medicine from Trieste (Italy), Sarajevo (Bosnia and Herzegovina), Zagreb (Croatia), Belgrade (Serbia), Athens (Greece), and Skopje (North Macedonia). The working group consisted of seven medico-legal experts. The primary objective was not epidemiological estimation, but the analysis, comparison, and standardization of forensic workflows applied to fatal cases of abusive head trauma (AHT), with the aim of developing a reproducible and transferable workflow model (Table 1).

**Table 1:** Proposed standardized forensic workflow for the investigation of fatal cases of abusive head trauma in the pediatric population.

Phase	Activities	Recommendations
Pre-autopsy	Anamnesis	The authors recommend a thorough collection of the decedent's medical history (e.g., child health booklet) in order to exclude pre-existing conditions potentially related to the cause of death. Whenever possible, the medical history of siblings should also be investigated, as it may suggest similar events of maltreatment or abuse. An assessment of the social context, with coordination with the appropriate social services can help yield important findings - when prior records are available.
	Scene Investigation	The authors strongly recommend the presence of a forensic pathologist at the scene. Particular attention should be paid to the search for medications and potential substances of abuse.
	Total-body CT scan	A total-body CT scan performed with a multi-slice scanner and thin slices is recommended to allow optimal three-dimensional reconstruction of skeletal structures and soft tissues.
	External Examination	The authors recommend a detailed description of any skin or mucosal lesions, malformations, and all relevant findings (e.g., conjunctival petechiae). Particular attention should be paid to burns, scars, and lesions suggestive of maltreatment or abuse. The external examination must be accompanied by comprehensive photographic documentation.
	Measurements	Body length and weight should be recorded. The following measurements are also recommended: head circumference, biparietal diameter, interpupillary distance, distance between the medial canthi, chest circumference, abdominal circumference, femur length, foot length, and vertex-to-sacrum distance.
	Head and Spine	The presence of hemorrhagic infiltrates of the scalp and galea aponeurotica should be described. The skull vault should be examined for fractures, whose course and length must be documented. When possible, access to the cranial cavity should be performed along cranial sutures (butterfly approach), with careful photographic or video documentation of traumatic lesions. The authors recommend fixation of the brain in formalin for subsequent detailed macroscopic examination. Examination of the spinal cord may be performed via an anterior or posterior approach. When feasible, removal of the spinal cord en bloc with the brain is recommended. Both eyeballs, together with the optic nerves, should be collected. For optimal sampling, formalin fixation is recommended.
Autopsy	Brain	After formalin fixation, the brain should be sectioned using serial coronal slices at approximately 1–1.5 cm intervals (Ludwig's scheme).
	Other Injuries	A layer-by-layer anatomical dissection is recommended to visualize and describe potential injuries to the thoracic and abdominal walls. All findings should be photographically documented, described, and measured.
	Sampling	One or more samples of major traumatic lesions should be collected to support the assessment of vitality and, when necessary, injury timing.
	Histological Examination	Routine staining (hematoxylin–eosin) is recommended. For brain lesions, special stains (e.g., Perls) and immunohistochemical stains (e.g., β-APP and Glycophorin-A) should be performed.
Post-autopsy	Toxicological Analysis	The authors recommend collection of peripheral blood, urine, vitreous humor and gastric content.
	Genetic Analysis	A sample of central blood and a spleen sample should be collected.

The workflow is structured into three sequential phases—pre-autopsy, autopsy, and post-autopsy—covering the entire medico-legal diagnostic process, from initial data collection and scene investigation to comprehensive autopsy examination and post-mortem histopathological assessment, with the aim of ensuring diagnostic accuracy and methodological standardization.

### 2.2 Case selection

Each participating institute independently conducted a retrospective search of its institutional database to identify fatal cases of AHT in the pediatric population. Inclusion criteria were: 1) age between 0 and 2 years; 2) death attributable to abusive head trauma or non-accidental head injury; 3) availability of complete medico-legal documentation, including autopsy reports and ancillary investigations.

Only cases from the last five years were included to ensure alignment with current diagnostic practices. Case selection was functional to workflow reconstruction and analysis, rather than quantitative evaluation.

### 2.3 Workflow reconstruction and analytical framework

Each center reconstructed its institutional forensic workflow based on real case management. A standardized analytical grid was developed to allow systematic comparison across institutions. The grid included pre-autopsy investigations (clinical history, scene investigation, imaging), autopsy procedures (external examination, internal dissection, sampling), post-autopsy analyses (histology, toxicology, ancillary tests), documentation practices (photographic and written reporting).

Each workflow was analyzed according to the following criteria: completeness (presence or absence of steps), timing (sequence of procedures), standardization (protocol-driven vs. discretionary practices), diagnostic contribution.

### 2.4 Standardized dataset and data comparability

To ensure comparability between centers, a shared minimum dataset was developed in the form of a structured spreadsheet. This dataset defined the essential variables to be collected in all cases, organized into three phases:

Pre-autopsy variables

- Clinical and anamnestic data (age, sex, medical history)
- Family context (including siblings)
- Scene investigation findings
- Radiological investigations (e.g., total-body CT)

Autopsy variables

- External examination findings and measurements
- Cranial, cerebral, spinal, and ocular findings
- Presence and type of traumatic lesions
- Sampling procedures (histology)

Post-autopsy variables

- Histological findings (routine and special stains)
- Toxicological analyses
- Genetic investigations (if performed)
- Differential diagnosis and exclusion of alternative causes

This standardized dataset ensured equivalence of collected data, allowing meaningful comparison across institutions.

### 2.5 Comparative analysis and consensus process

A structured comparative analysis was performed using the standardized dataset and analytical framework.

The process followed a modified Delphi-like consensus methodology, consisting of iterative expert discussions, identification of shared practices and discrepancies, detection of missing or inconsistently collected data and progressive refinement of procedural steps.

The objective was to define mandatory procedures (minimum reproducible standard) and recommended procedures (quality improvement level).

### 2.6 Gap analysis and identification of missing variables

The comparative analysis revealed significant heterogeneity in documentation and practice. Frequently missing elements included standardized scene investigation data, systematic ophthalmological examination, spinal cord evaluation, toxicological screening, structured documentation of differential diagnosis.

These gaps informed the development of an expanded workflow and dataset, including variables not consistently present in institutional records but considered essential according to current literature.

### 2.7 Integration with international literature

The workflow model was developed in alignment with current international literature and existing recommendations. Particular attention was given to the role of pre-autopsy imaging and scene investigation, the necessity of a complete autopsy including brain, spine, and ocular examination, the importance of histological and immunohistochemical analyses, the need for systematic exclusion of alternative causes of death.

Literature sources were used as a benchmark to validate and refine the proposed workflow.

### 2.8 Development of the standardized workflow model

Based on the comparative analysis, gap identification, and literature integration, a standardized workflow model was developed. The workflow is structured into three sequential phases: 1) Pre-autopsy, 2) Autopsy, 3) Post-autopsy.

For each phase, procedures were categorized as mandatory (minimum standard for reproducibility) and recommended (enhanced diagnostic accuracy).

### 2.9 Development of operational tools

The final methodological output consists of three integrated tools: 1. Workflow chart – defining the sequence of forensic actions; 2. operational checklist – ensuring systematic execution and documentation; 3. standardized dataset (spreadsheet) – ensuring comparability across institutions.

These tools were designed to be practical, reproducible, and compatible with routine forensic practice.

### 2.10 Ethical considerations

All data were analyzed in anonymized form. The study was conducted in accordance with national regulations governing medico-legal investigations and did not involve experimental procedures beyond standard forensic practice.

## 3 Workflow proposal

From the working group discussions, emerged the need to divide the forensic diagnosis into three sequential phases.

### 3.1 Pre-autopsy stage

- Conduct a thorough anamnesis, including, when present, the clinical history of siblings.
- Perform a scene investigation in collaboration with the Judicial Authority.
- Perform a total body CT scan.

### 3.2 Autopsy stage

- Post-mortem examination documented with photographs
- Collect toxicological samples
- Adopt a layer-by-layer internal examination documented with photographs
- Access the brain using the proper approach depending on the age of the child, and proceed with fixation of the entire brain.
- Remove and proceed with fixation of the entire spine.
- Collect and proceed with fixation of both eyes.
- Take complete histological samples from all relevant tissues.

### 3.3 Post-autopsy stage

- Process histological samples using standard staining techniques. Consider additional stains, including Perls’ stain and relevant immunohistochemistry, as well as beta-PP (*β*-PP) analysis.
- Exclude alternative causes of death through both macroscopic and microscopic evaluation.

## 4 Discussion

The aim of forensic medicine is to assist legal proceedings by determining the causal relationship between an action and its consequences (2, 12). Unlike other medical disciplines, forensic medicine is often characterized by the lack of a structured evidence-based practice or protocols (2, 12–14). As with any other medical field, forensic medicine is not exempt from error (14), and an increasing importance is being placed on the development of and adherence to guidelines and best practices (15).

The working group focused its attention on both European and Balkan areas initially reviewing current guidelines. Recent attention to this subject is reflected in the literature. In 2025, the European Council of Legal and Forensic Medicine (ECLFM) formally highlighted the need for guidelines on the examination of child abuse victims (15). When individual national contexts were considered, substantial differences emerged. Unlike France (11, 16–18), Italy for example does not have specific national guidelines for the management of abusive head trauma (AHT). Instead, several protocols and recommendations have been developed independently by individual referral centers. The main Italian reference is the 2018 study by Da Dalt et al. (19), which provides recommendations to guide clinicians in the diagnostic process (19). Additional documents have been published by the Italian Society of Neonatology (SIN) (20), the Italian Society of Pediatric Emergency-Urgency Medicine (SIMEUP) (21), the Italian Coordination of Services against Child Abuse and Neglect (22), and the Bambino Gesù Pediatric Hospital (23). The challenge becomes even greater when attempting to identify sources that can guide medico-legal diagnosis and autopsy practice in fatal cases, as the available literature in this specific field remains limited (9, 10). A similar scenario can be observed in countries of the central Balkan region, such as Serbia, where no national guidelines are currently available. Furthermore, recent studies on cranial trauma excluded cases involving violent mechanisms (24).. The same situation applies to Greece, Croatia, Bosnia and North Macedonia, where medico-legal practice largely relies on international literature.

The idea underlying the discussion among the professionals involved arose precisely from the need to jointly develop a practical, simple, yet comprehensive protocol. The intention was to give the research a practical orientation, allowing it to be applied to everyday practice, with the aim of making the study reproducible and comparable across different settings, as well as more accessible for use in court.

Comparing workflows used in different countries not only allowed analysis of strengths and weaknesses of individual methodologies, but also highlighted procedural differences from the earliest stages of investigation. These differences concerned, for example, the acquisition of photographic documentation, which is considered of great importance for documenting injuries (10), or the use of toxicological and histological investigations, which are sometimes considered non-essential, particularly when obvious macroscopic findings are present.

Pre-autopsy radiological examination, including the eye examination through Optical coherence tomography (OCT) (9) is now considered of fundamental importance in the investigation of AHT (2, 10, 25). It plays a key role in the evaluation of possible fractures and cranial injuries and also providing valuable information for lesion dating (10). Toxicological analyses were incorporated into the checklist, despite their limited emphasis in the latest post-mortem diagnostic literature, especially to exclude potential intoxication by medications or other substances subject to misuse.

Given the fundamental necessity of performing a full autopsy (8, 9, 11), particular attention has been directed toward several specific aspects. In accordance with the most recent literature, an accurate ophthalmological examination is strongly recommended (8). The limited amount of available literature, especially regarding post-mortem evaluation of ocular hemorrhages (26–28), represents a significant limitation. Nevertheless, the removal of both eyes remains of fundamental importance (2, 8). This can be performed through an anterior approach when examination of surrounding orbital structures is not required, or through a posterior approach when a complete assessment of the orbital contents is necessary - including periocular fat, the optic nerve, and the nerve sheaths (10, 29). In addition to the assessment of the cranial vault and the brain (2, 10) to assess the hallmark signs (10), the examination of the spinal cord cannot be disregarded and should be considered an integral part of the post-mortem investigation (2, 9, 29). For this reason, spinal cord examination has been included in the proposed checklist, in line with current literature considerations (30, 31).

Finally, forensic pathology cannot disregard histological examination of the collected samples (9, 10). In addition to routine hematoxylin and eosin staining, the use of Perls’ histochemical staining is recommended for the detection of hemosiderin deposits (28). In accordance with the literature, the checklist also proposes the use of immunohistochemistry. In particular, beta-amyloid precursor protein (*β*-APP) staining is useful for the early detection of axonal injury, while Glycophorin-A staining allows evaluation of hemorrhagic areas (9, 10, 32).

Attention is also drawn to another critical aspect of autopsy diagnosis in AHT, namely the identification of possible alternative causes that may explain the observed findings. The importance of differential diagnosis is emphasized in the European context by the most recent updates of the French guidelines (18) and by current international literature (33–35). Given the extensive number of potential differential diagnoses (36), excluding all alternative conditions is often challenging. This is particularly true in deceased patients born at term with minimal or no prior hospitalizations, and in the absence of comprehensive hematological, genetic, or other specialized investigations.

## 5 Limitations

This proposal has several limitations that should be considered. Firstly, this study has inherent methodological limitations related to the retrospective design, as case selection was constrained by the availability of existing data rather than predefined inclusion criteria. Moreover, the conclusions drawn from the cases could not be supported by quantitative validation and were mainly based on qualitative or interpretative observations. The expert working group understands that this is a proposal and a simple starting point to address a complex medico-legal problem.

## 6 Conclusion and future directions

In order to ensure the accuracy of the diagnosis, the workflow proposal was designed to promote a unified approach among professionals and to maintain a high standard of quality. Efforts were made to include only procedures and assessments that can be routinely performed, creating a practical checklist for the management of these cases in routine forensic practice. This approach is expected to ensure greater uniformity in working methods and, at the same time, generate a consistent and shared dataset useful in daily forensic practice. In future, our goal is to create a Balkan registry for AHT cases based on a model used for an ongoing project on sudden cardiac death (37). Further meetings will be conducted to address the standardization of heterogeneous medico-legal practices across the Balkan region.

## Data Availability

The data analyzed in this study is subject to the following licenses/restrictions: The dataset is in the availability of the corresponding author and available if requested. Requests to access these datasets should be directed to sderrico@units.it.
